# Comparison of Stool Microbiome in Children with Cystic Fibrosis Treated with and Without Elexacaftor–Tezacaftor–Ivacaftor—A Pilot Study †

**DOI:** 10.3390/ijms27020814

**Published:** 2026-01-14

**Authors:** Senthilkumar Sankararaman, Ruitao Liu, Xinyu Sun, Mauricio Retuerto, Terri Schindler, Erica Roesch, Thomas J. Sferra, Mitch Drumm, Mahmoud Ghannoum, Liangliang Zhang

**Affiliations:** 1 Division of Pediatric Gastroenterology, Rainbow Babies and Children’s Hospital, Cleveland, OH 44106, USA; thomas.sferra@uhhospitals.org; 2 Department of Pediatrics, Case Western Reserve University School of Medicine, Cleveland, OH 44106, USA; erica.roesch@uhhospitals.org; 3 Department of Population and Quantitative Health Sciences, Case Western Reserve University School of Medicine, Cleveland, OH 44106, USA; rxl761@case.edu (R.L.); xxs410@case.edu (X.S.); lxz716@case.edu (L.Z.); 4 Center for Medical Mycology, Department of Dermatology, Case Western Reserve University School of Medicine, Cleveland, OH 44106, USA; retuertomauricio@gmail.com (M.R.); mag3@case.edu (M.G.); 5 Division of Pediatric Pulmonology, Rainbow Babies and Children’s Hospital, Cleveland, OH 44106, USA; terri.schindler@uhhospitals.org; 6 Department of Genetics and Genomic Sciences, Case Western Reserve University School of Medicine, Cleveland, OH 44106, USA; mxd34@case.edu; 7 Department of Dermatology, University Hospitals Cleveland Medical Center, Cleveland, OH 44106, USA

**Keywords:** gut microbiome, microbiota, cystic fibrosis (CF), dysbiosis

## Abstract

Prior studies in people with cystic fibrosis (CF) demonstrated a positive impact of ivacaftor on the stool microbiome. However, studies evaluating the impact of elexacaftor–tezacaftor–ivacaftor (ETI) on gut dysbiosis are limited. In this prospective, observational study, we evaluated the differences in stool microbiome in children (aged 2–17 years) with CF who were treated with ETI for at least two months and compared with children with CF who did not receive ETI. We also included healthy siblings as controls. There were no significant differences in the demographics between the groups. There were no significant differences in alpha diversity between the groups for both bacteriome and mycobiome. Alpha diversity showed a negative trend with the duration of ETI therapy for both bacteriome and mycobiome. *Firmicutes* and *Proteobacteria* were the most abundant phyla and core members across all samples, regardless of disease status or treatment. *Ascomycota* and *Basidiomycota* were the most abundant and core members across all samples, regardless of disease status or treatment. Alpha diversity showed a negative trend with the duration of ETI therapy for both bacteriome and mycobiome in children with CF treated with ETI. Future studies are needed to confirm or refute our preliminary findings.

## 1. Introduction

Cystic fibrosis (CF) transmembrane conductance regulator (CFTR) is an anion channel, and its dysfunction in CF leads to the accumulation of thick, acidic, dehydrated mucus in epithelial tissues of various organs. People with CF have multisystem involvement, including the respiratory tract, gastrointestinal tract, pancreas, liver, sweat glands, vas deferens, etc. The respiratory tract accounts for much of the mortality and morbidity in CF. Currently, at least 92% of people with CF are either receiving or eligible for CFTR-directed modulator therapies, and the majority of them (76%) were treated with elexacaftor–tezacaftor–ivacaftor (ETI, Trikafta^®^) per the 2024 CF Foundation Patient Registry Report [1]. Altered microbial milieu in the respiratory tract is well documented, and changes accompanying respiratory microbiota after modulator therapy are better researched [2,3,4,5,6,7]. This improvement in the lung microbiome occurs as early as a month after initiating ETI [8].

Similarly to the airway, people with CF have intestinal microbial dysbiosis. Intestinal dysbiosis in CF likely occurs for several reasons, such as CFTR dysfunction leading to an altered and acidic inflammatory intestinal milieu, exocrine pancreatic insufficiency, a diet high in calorie-dense processed foods, and frequent use of medications such as antibiotics [9,10,11]. Gut dysbiosis in CF has been linked to many local and systemic implications. Enaud and colleagues noted an inflammatory bowel disease (IBD)-like microbial dysbiosis in CF characterized by dominance of *Staphylococcus*, *Streptococcus*, and *Veillonella dispar* [12]. Proinflammatory gut dysbiosis could also predispose individuals to colon cancer, and people with CF have an increased risk of colon cancer [13,14]. Systemically, gut dysbiosis in CF has been associated with pulmonary exacerbations and liver involvement, which are referred to as the gut–lung axis [15] and gut–liver axis, respectively [2,16,17].

Many studies have demonstrated that people with CF have dysbiosis characterized by a decrease in gut microbial diversity (both a decrease in richness and abundance) and reduced abundance of beneficial bacteria [18,19]. The first modulator, ivacaftor, which was approved in 2012, has shown improvement in the gut microbial diversity [20,21]. However, ivacaftor was available only to individuals with specific and relatively rare CF mutations. ETI was first approved in 2019 for adults with the most common genetic mutation (F508del) in CF, and in 2023, it was approved for children above two years of age. The effect of ETI on the gut microbiome (bacteriome) is not well studied, and mycological studies are even rarer [2,9]. In this pilot study, we aimed to evaluate the effect of ETI on the gut microbiome in children (between 2 and 17 years) with CF. Our hypothesis was that ETI may modulate the gut microbiome, resulting in changes in the diversity of both gut bacteriome and mycobiome. This paper is an extended version of our paper published in the 2023 Annual meeting, North American Society for Pediatric Gastroenterology, Hepatology and Nutrition, Abstract No. 705/782 [22].

## 2. Results

### 2.1. Demographics and Clinical Characteristics

We included 31 children (12 CF with ETI treatment, 12 CF without ETI treatment, and 7 non-CF siblings). One sample in the 12 CF without ETI treatment was excluded due to suboptimal quality, resulting in 30 patients for analysis. No significant differences between the groups have been identified with respect to age, sex, and other pertinent clinical comorbidies (Table 1)

In our entire cohort, there were no patients with malnutrition, cystic fibrosis-related liver disease (CFLD), or cystic fibrosis-related diabetes (CFRD). Similarly, no patients were receiving probiotics.

### 2.2. Bacteriome Analysis

For bacteriome analysis, 16S rRNA sequencing was used (refer to the Section 4 Materials and Methods and Supplementary Materials for further information).

#### 2.2.1. Richness and Relative Abundance

**Figure 1:**
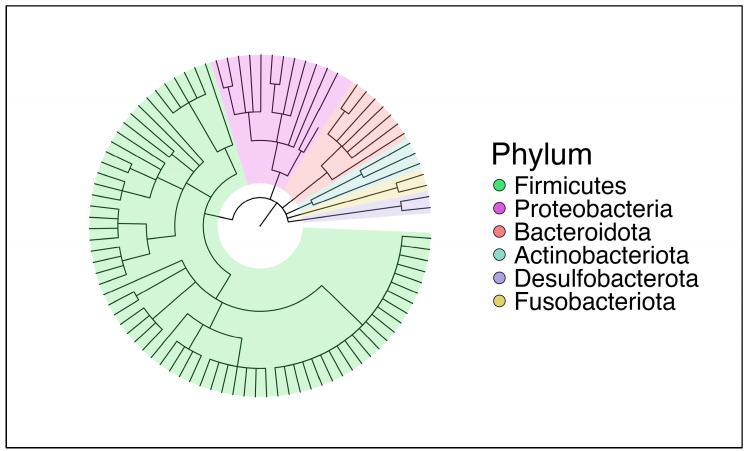
Taxonomy distribution of bacterial phyla in the entire cohort.

In a circular taxonomic tree (cladogram, generated using the *MicrobiomeMarker* package (v1.10.0), the overall taxonomic diversity of the entire dataset was represented in biological hierarchies (Figure 1). The tree is color-coded at the phylum level, allowing visualization of the dominant bacterial groups. By genus richness, *Firmicutes* was the most diverse phylum, followed by *Proteobacteria*. *Bacteroidota* and *Actinobacteriota* also held notable representation. *Desulfobacterota* and *Fusobacteriota* appeared less abundant, occupying fewer branches in the cladogram.

**Figure 2:**
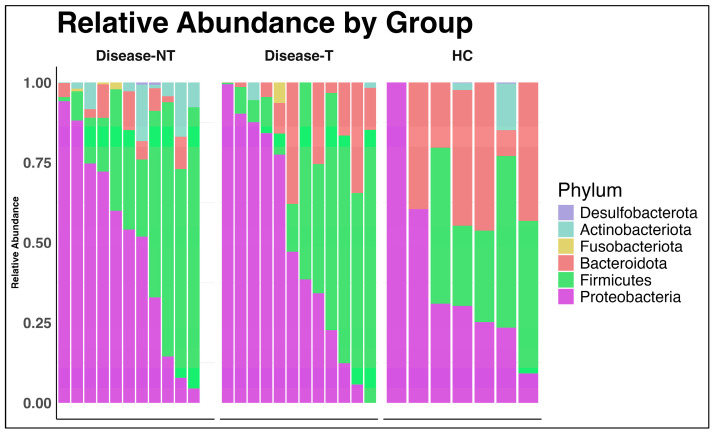
Relative abundance of bacterial phyla in the three groups (Disease-NT—Children with cystic fibrosis not treated with Elexacaftor–Tezacaftor–Ivacaftor; Disease-T—Children with cystic fibrosis treated with Elexacaftor–Tezacaftor–Ivacaftor; HC—Healthy sibling controls).

*Proteobacteria* and *Firmicutes* were the most abundant and core members across all samples, regardless of disease status or treatment (Figure 2). The abundance of *Bacteroidota* was relatively higher in the healthy controls compared to the CF groups.

#### 2.2.2. Alpha Diversity

**Figure 3:**
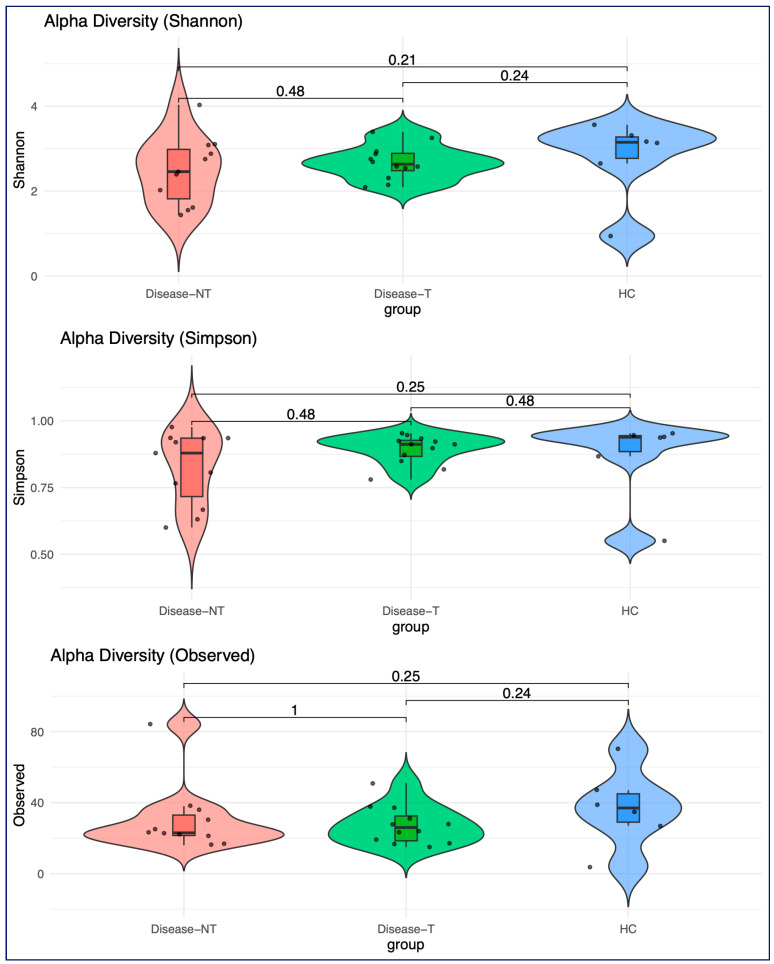
Comparison of bacterial alpha diversity across all three groups using three alpha diversity metrics (Shannon’s index, Simpson’s index, and observed alpha diversity). (Red, Disease-NT—Children with cystic fibrosis not treated with Elexacaftor–Tezacaftor–Ivacaftor (red color); Disease-T—Children with cystic fibrosis treated with Elexacaftor–Tezacaftor–Ivacaftor (green color), and HC—Healthy sibling controls (blue)).

We did rarefaction before exploring the diversity, selecting an inclusion of 500 sequencing depths as our criteria (Supplemental Figure S1 and Supplemental Table S1). We excluded one more sample from the control group, and this led to 29 patients (12 CF with ETI treatment, 11 CF without ETI, and 6 non-CF siblings). Overall, the results suggested that people with CF were associated with a slight reduction in microbial diversity across all three alpha diversity metrics (Shannon’s index, Simpson’s index, and observed diversity) when compared to healthy controls (Figure 3). ETI treatment appeared to partially restore the alpha diversity, but the differences between groups were not statistically significant.

**Figure 4:**
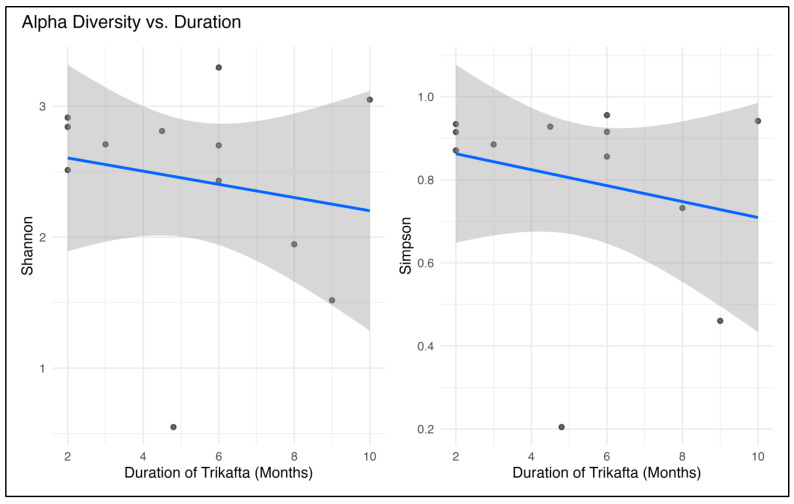
Relation between alpha diversity (bacterial) and duration of Elexacaftor–Tezacaftor–Ivacaftor (Trikafta^®^) treatment.

A flexible regression method (generalized additive modeling (GAM)) was used to see whether the length of ETI therapy was associated with changes in microbial diversity. Alpha diversity showed a negative trend against the duration of ETI therapy (Figure 4). For both Shannon’s index and Simpson’s index, the *p* was <0.001. However, the performance of GAM was suboptimal, which is likely due to the smaller sample size in our cohort (Supplemental Figure S2).

#### 2.2.3. Beta Diversity

**Figure 5:**
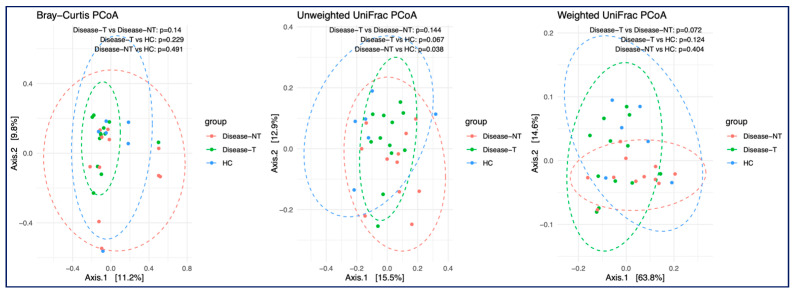
Comparison of bacterial beta diversity across all three groups. From left to right, three measurements were used, including Bray–Curtis, unweighted UniFrac, and weighted UniFrac distance matrices. (Red, Disease-NT—Children with cystic fibrosis not treated with Elexacaftor–Tezacaftor–Ivacaftor (red color); Disease-T—Children with cystic fibrosis treated with Elexacaftor–Tezacaftor–Ivacaftor (green color); and HC—Healthy sibling controls (blue)). The PCOA plot based on weighted UniFrac distance matrices indicated that Axis 1 accounted for 63.8% of the variation. The accuracy of this contribution value was verified, and we used the function in the ape R package (v4.4.1) to double-check the validity.

Overall, the beta diversity (Bray–Curtis and weighted UniFrac Principal Coordinate Analysis (PCoA) between the groups was not significant (Figure 5). The unweighted UniFrac PCoA (middle panel) showed a significant difference in microbial composition between CF children not treated with ETI and healthy controls (*p* value—0.038). As unweighted UniFrac PCoA incorporated the phylogenetic information but considered only the presence or absence of taxa, not their abundance and this could be a reason for this significant difference in *p* value noted here. Also, in unweighted UniFrac PCoA, the ETI treatment group demonstrated an intermediate position between the untreated group and the healthy control group, with a *p*-value of 0.067 when compared to healthy controls, indicating a possible trend toward microbial restoration with ETI treatment.

#### 2.2.4. Differential Analysis

**Figure 6:**
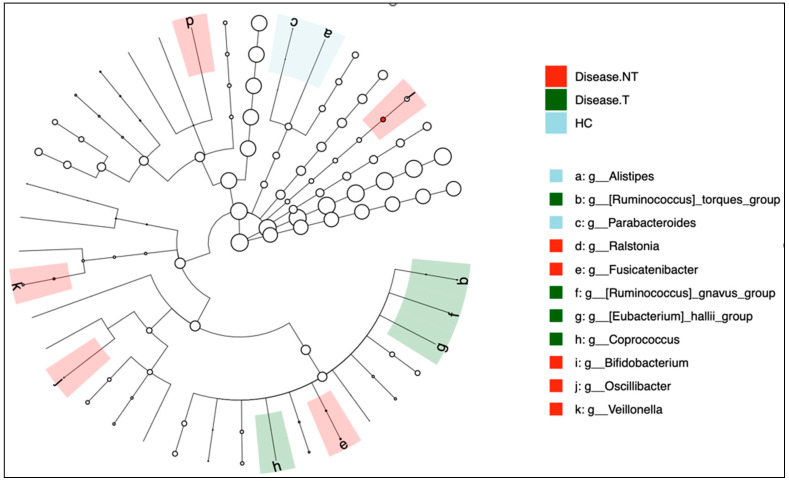
The cladogram highlighted differentially abundant bacterial genera identified by DESeq2; branches are shaded by the group of enrichment. Node size scales with relative abundance (Red, Disease-NT—Children with cystic fibrosis not treated with Elexacaftor–Tezacaftor–Ivacaftor (red color); Disease-T—Children with cystic fibrosis treated with Elexacaftor–Tezacaftor–Ivacaftor (green color), and HC—Healthy sibling controls (blue)).

The differential analysis was noted in the cladogram displaying the significant genus selected by DESeq2 (Figure 6). In the cladogram, color shading indicated that the group in which each genus was significantly enriched. Taxa highlighted in red, green, or blue correspond to genera that demonstrated selective enrichment in Disease-NT (children with CF not treated with ETI), Disease-T (children with CF treated with ETI), or HC (healthy sibling controls), respectively. These colors allowed visual tracing of group-associated differences along the phylogenetic tree. Branches or nodes without color indicated that the taxa did not differ significantly between groups.

In this cohort, the notable and clinically pertinent genera were *Alistipes* and *Bifidobacterium*. *Alistipes* was abundant in the controls, *p* < 0.01. *Bifidobacterium* was relatively abundant in the children with CF not treated with ETI and in healthy controls, *p* < 0.01. In Supplemental Figure S3—Cladogram of all-level significance were noted using DESeq2. Supplemental Figures S4 and S5 illustrated a heat map and a bar plot, respectively, by DESeq2 analysis. These findings were further reiterated by LEfSe analysis. 

### 2.3. Mycobiome Differences

We used internal transcribed spacer (ITS) sequencing for mycobiome analysis and included all 31 patients (12 CF with ETI treatment, 12 CF without ETI treatment, and 7 non-CF siblings). With the addition of one more patient in the CF without ETI treatment group (12 here instead of 11 in the bacteriome analysis, we did not find any significant statistical differences between the three groups for demographic and clinical characteristics (findings similar to Table 1).

#### 2.3.1. Richness and Relative Abundance

**Figure 7:**
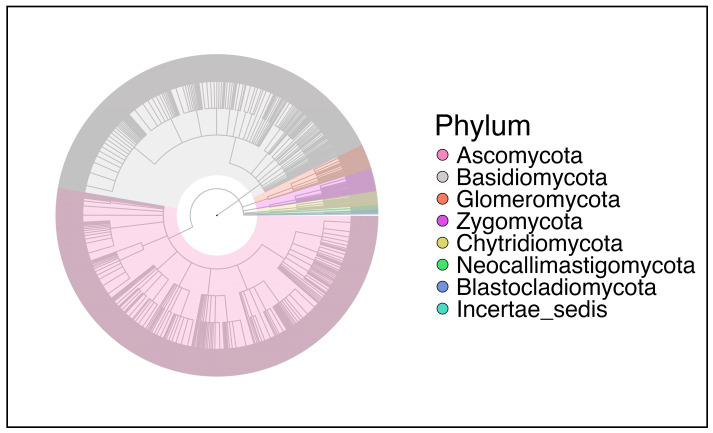
Taxonomy distribution of all fungal phyla in the entire cohort.

The broadest phylum was *Ascomycota*, followed by *Basidiomycota* with the highest number of genera in them (Figure 7). *Glomeromycota*, *Zygomycota*, *Chytridiomycota*, *Blastocladiomycota*, and *Neocallimastigomycota* had reduced richness, occupying fewer branches in the tree.

**Figure 8:**
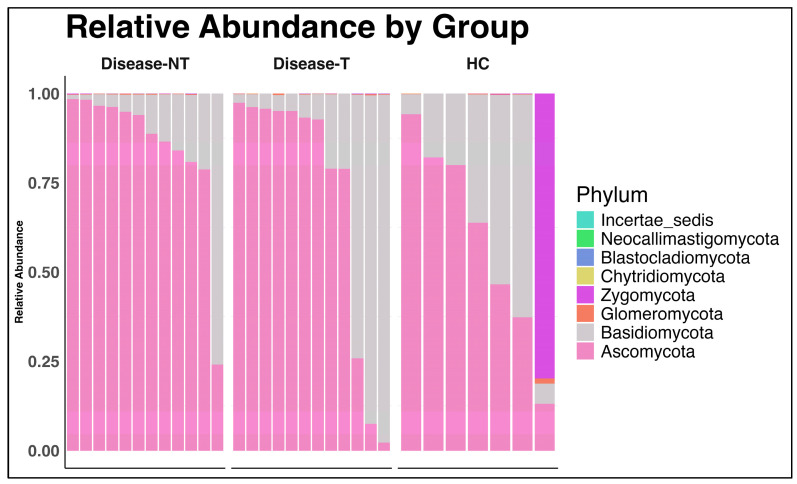
Relative abundance of fungal phyla in the three groups. (Disease-NT—Children with cystic fibrosis (CF) not treated with Elexacaftor–Tezacaftor–Ivacaftor (ETI); Disease-T—Children with CF treated with ETI; HC—Healthy sibling controls).

*Ascomycota* and *Basidiomycota* were the most abundant and core members across all samples, regardless of disease status or treatment (Figure 8). *Ascomycota* was more abundant in the CF patients (both with and without ETI), with a decreased *Basidiomycota*/*Ascomycota* ratio than in the controls.

We calculated the *Basidiomycota*/*Ascomycota* ratio in all three groups. The *Basidiomycota*/*Ascomycota* ratio was higher in the healthy control compared to the CF groups (Supplemental Figure S6). Significant difference was noted between the healthy control group and the CF group without ETI treatment (*p* value 0.028). However, the *Basidiomycota*/*Ascomycota* ratio comparison between the two CF groups or the CF group with ETI treatment and the healthy group was not significant.

#### 2.3.2. Alpha Diversity

**Figure 9:**
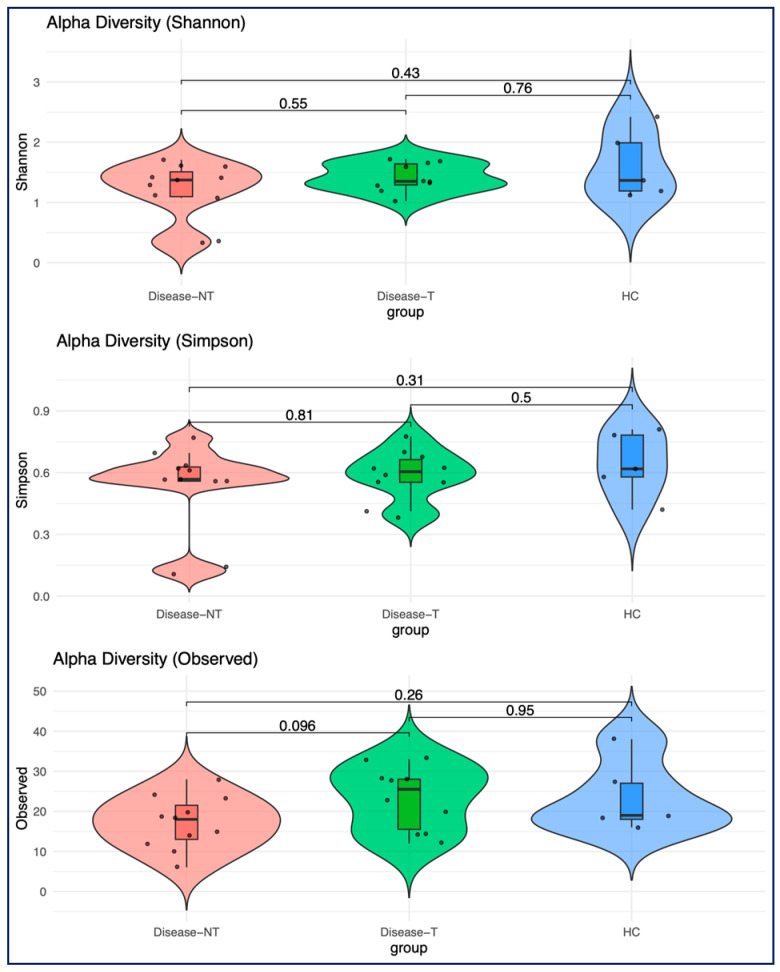
Comparison of fungal alpha diversity across all three groups. From top to bottom, three different indices were applied to calculate the alpha diversity—Shannon, Simpson, and the Observed index. (Red, Disease-NT—Children with cystic fibrosis not treated with Elexacaftor–Tezacaftor–Ivacaftor (red color); Disease-T—Children with cystic fibrosis treated with Elexacaftor–Tezacaftor–Ivacaftor (green color), and HC—Healthy sibling controls (blue)).

We did rarefaction before we explored the diversity, selecting an inclusion of 500 sequencing depths as our criteria. The effect of rarefaction thresholds using rarefaction curves and sample sequencing depth distribution were noted in Supplementary Figure S7 and Supplemental Table S2, respectively. After quality control, 10 patients in the CF treated with ETI, 11 patients in the CF without ETI, and 5 patients in the health control were included for final analysis for diversity. No differences in alpha diversity were noted between the three groups across all different alpha diversity metrics (Shannon’s index, Simpson’s index, and the observed diversity) (Figure 9).

**Figure 10:**
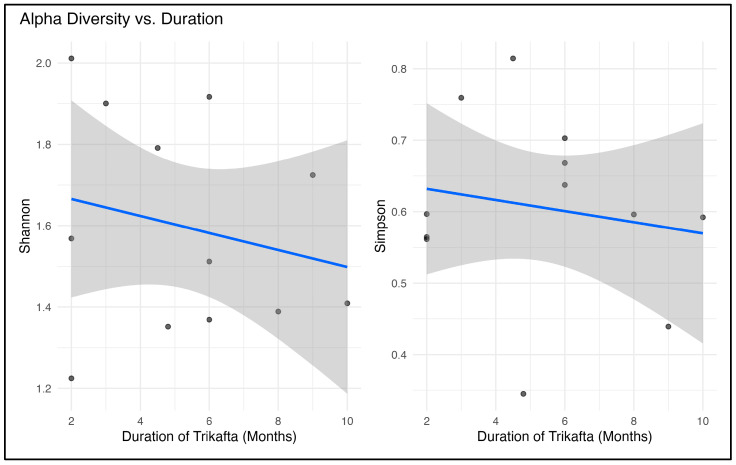
Relation between fungal alpha diversity and duration of ETI (Trikafta^®^) treatment.

We calculated the relation between alpha diversity and duration of ETI treatment. We noted a negative trend (*p* value < 0.01 for both Shannon index and Simpson index) (Figure 10). The performance of GAM was illustrated in Supplemental Figure S8.

#### 2.3.3. Beta Diversity

**Figure 11:**
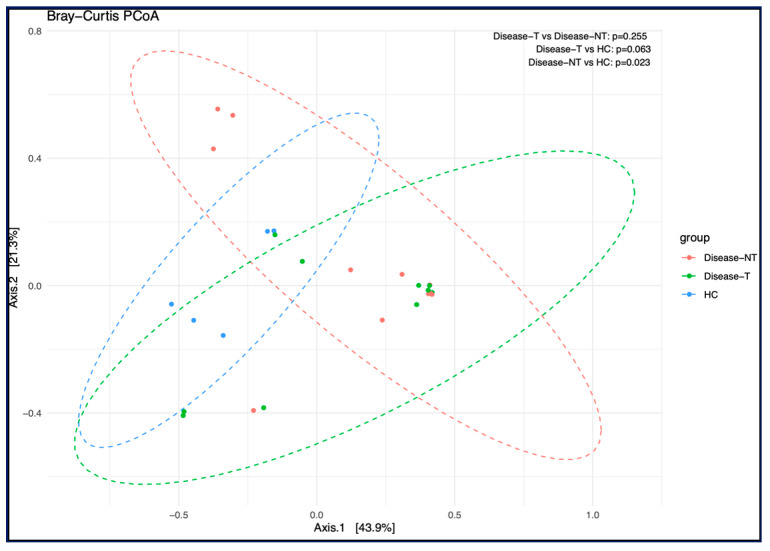
Comparison of fungal beta diversity across all three groups. (Red, Disease-NT—Children with cystic fibrosis not treated with Elexacaftor–Tezacaftor–Ivacaftor (red color); Disease-T—Children with cystic fibrosis treated with Elexacaftor–Tezacaftor–Ivacaftor (green color), and HC—Healthy sibling controls (blue)).

Overall, the beta diversity (weighted and unweighted UniFrac PCoA between the groups was not significant (data not included). However, with Bray–Curtis PCoA, the beta diversity between CF children not treated with ETI and healthy controls was significant, with a *p* value—0.023 (Figure 11).

### 2.4. Functional Microbial Differences

**Figure 12:**
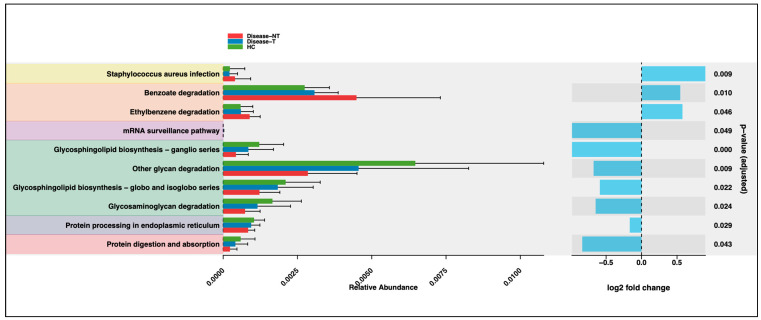
Functional differences between the three groups (noted with fold changes and *p* values).

Functional composition was inferred with PICRUSt2, yielding a sample-by-function abundance matrix. Group differences were then tested and visualized with our ggpicrust2 R package [23]. Figure 12 displayed the functional differences (fold change) in some of the core pathways between the three groups. Children with CF treated with ETI occupied an intermediate status between healthy control group and children with CF who did not receive ETI. In Figure 12, the pairwise *p*-values were noted between children with CF not treated with ETI and healthy sibling controls as there were significant changes between them. The *Staphylococcus aureus* infection module was more abundant in CF children not treated with ETI than in the other two groups. Benzoate and ethylbenzene degradation (representing xenobiotic metabolism) were enriched in CF children not treated with ETI. The other pathways were enriched in healthy controls. The three modules, *Staphylococcus aureus* infection, benzoate degradation, and ethylbenzene degradation were noted as a positive fold change, and the rest of the pathways that were enhanced in healthy controls were noted as a negative fold change.

Supplemental Table S3 demonstrated the pairwise *p*-values between all the groups and the fold changes (in z score) were noted in Supplemental Figure S9.

## 3. Discussion

In this prospective observational pilot study, we compared the gut microbiome of children with CF who were treated with ETI for at least two months vs. CF children who did not receive ETI. The demographic and clinical characteristics between the two CF groups (treated with and without ETI) were not significantly different. There were no significant differences in the demographics between the groups. We also included seven non-CF siblings as healthy controls. There were no significant differences in alpha diversity between the groups for both bacteriome and. mycobiome. Overall, alpha diversity showed a negative trend with the duration of ETI therapy for both bacteriome and mycobiome in children with CF treated with ETI.

Even though there was no significant differences in alpha diversity between the groups, in the bacteriome, alpha diversity in the CF population was slightly lower than healthy controls. Similarly to this study, lower alpha diversity in children with CF was noted in prior studies compared to healthy controls [17,24,25,26,27]. Previously, ivacaftor has been shown to improve gut microbial diversity [20,21]. On the contrary, other studies did not show significant improvement in diversity and richness after single (ivacaftor) or dual modulators (lumacaftor/ivacaftor and tezacaftor/ivacaftor therapy) [28,29,30]. We observed that ETI treatment appeared to partially restore the alpha diversity, but the differences between three groups were not statistically significant. A smaller sample size could be one of the reasons for the lack of significant differences in alpha diversity between three groups (particularly between the CF group without ETI therapy and the non-CF sibling group) in our study.

In our cohort, the alpha diversity showed a significant negative trend with the duration of ETI therapy, and findings from the PROMISE study also reiterated our observation [31]. The PROMISE study included 345 samples from 124 participants with CF ≥12 years old recruited from 18 accredited CF centers [31]. Many studies evaluated the relationship between ETI and gut microbial changes in a longitudinal fashion. In the PROMISE study, alpha diversity after six months of therapy was significantly lower than one month of therapy and also pre-ETI [31]. In the preliminary report, results from the RECOVER study from Ireland analyzed 179 stool samples from 106 participants (64 samples at baseline, 57 at 1 month, 58 at 6 months) [32]. They noted a significant increase in alpha diversity (Shannon) between baseline and 1 month of ETI therapy for participants aged ≥ 12 years and but no changes were seen in the 6–11 years group [32]. Further, no relationship was noted between other microbiome characteristics and abdominal symptoms or gut inflammation. A study from Spain included 31 patients aged 6–18 years with CF [33]. After six months of ETI treatment, no significant changes in the alpha diversity were noted [33].

In a recent longitudinal study by Reasoner and colleagues, ETI therapy increased gut microbial diversity and richness, and reduced intestinal inflammation and antibiotic-resistance genes [34]. In a study involving 20 CF participants, alpha diversity gradually declined at 3 and 6 months but increased after 6 months [10]. Here, the authors postulated that the initial decrease in diversity was due to reflection of possible selection pressures within the bacterial community, secondary to physiological changes from ETI [10]. Knoll et al. demonstrated that the changes in the airway microbiome occurred rapidly after ETI initiation, but changes in the gut microbiome occurred more gradually [35]. No significant changes in gut microbiota were noted in the first three months, and changes started to emerge between 6 and 12 months of ETI therapy [35]. The variations in alpha diversity post-modulator therapy could be due to multiple factors such as age of the patient, methodology used, and duration of ETI.

Similarly to gut microbial diversity, changes in alpha diversity (both increase or decrease) in lung microbiota with the duration of ETI therapy have also been noted [8,36]. We noted a significant difference in beta diversity between CF children not treated with ETI and healthy controls (by unweighted UniFrac PCoA). The PROMISE study also noted distinct differences in microbiota composition at three timepoints (pre-ETI and one and three months post-ETI) (*p*-value = 0.001, by PERMANOVA) [31].

In our cohort, *Proteobacteria* and *Firmicutes* were the most abundant phyla and core members across all samples, regardless of disease status or treatment, and a similar pattern was noted in a study by Bastón-Paz et al. [37]. In a study by Gutiérrez-Díaz et al., *Actinobacteriota* and the *Firmicutes* were the most abundant phyla [33]. A reduction in *Actinobacteriota* phylum and an increase in the *Firmicutes* phylum abundance were documented after ETI treatment [33]. Reasoner et al. also noted a similar increase in *Firmicutes* after ETI [34]. We noted that the abundance of *Bacteroidota* was relatively higher in the healthy controls compared to the CF groups. This observation has also been noted by many prior CF studies [27,38,39,40,41,42].

Marsh et al. compared 20 CF patients (both adults and children) with 10 healthy controls and noted that administration of ETI beyond 17 months resulted in a shift in microbial composition towards healthy controls [10]. We noted that *Bifidobacterium* was highly abundant in the children with CF not treated with ETI and in healthy controls. With many CF studies demonstrating a reduction in *Bifidobacterium* in CF, Duytschaever et al. noted significant underrepresentation of *Bifidobacterium* in people with CF, similar to our observation [9,19]. Also, in our cohort, *Alistipes* was highly abundant in the controls compared to the CF patients. Prior studies have also noted paucity of *Alistipes* in people with CF [43]. We did not see an increase in Alistipes post-ETI. In contrast to our findings, Casey et al. noted that ETI therapy was associated with a significant increase in *Alistipes* post-ETI [44].

Studies involving gut mycobiome in people with CF remain sparse. In our mycobial analysis, *Ascomycota* and *Basidiomycota* were the most abundant and core members across all samples, regardless of disease status or treatment. Bastón-Paz also noted similar *Ascomycota* dominance in the gut in CF patients [37]. We noted no differences in alpha diversity between the three groups across all three alpha diversity metrics in the fungal mycobiome. However, with Bray–Curtis PCoA, the beta diversity between CF children not treated with ETI and healthy controls was significant. Zubiria-Barrera et al. evaluated the impact of antibiotic treatment on the naso-sinal and gut mycobiome in 12 people with CF and compared it with 38 healthy controls [45]. Even though the alpha diversity was lower in the CF group compared to the control population, no significant difference was observed between the gut mycobiome of the two cohorts [45]. Similarly, no difference in beta diversity was noted between the two groups [45].

The *Basidiomycota*/*Ascomycota* ratio was higher in the healthy control group compared to the two CF groups. Significant difference was noted between the healthy control group and the CF group without ETI treatment, with a higher ratio in healthy controls. However, the *Basidiomycota*/*Ascomycota* ratio comparison between the other two groups (comparing CF group with ETI treatment and the healthy control group or comparing both CF groups) were not significant. The lower *Basidiomycota*/*Ascomycota* ratio in CF is likely due to increased representation of *Ascomycota* (many fungal pathogens belong to *Ascomycota*) in CF. A significantly altered *Basidiomycota*/*Ascomycota* ratio may be indicative of gut dysbiosis and prior studies noted that an increased *Basidiomycota*/*Ascomycota* ratio in inflammatory bowel disease and colon cancer and a decreased ratio in cirrhosis [46,47,48,49]. Further, the predominance of *Ascomycota* in respiratory mycobiota in CF with a lower *Basidiomycota*/*Ascomycota* ratio is well known, but studies are lacking in the CF gut mycobiome [50,51,52]. To the best of our knowledge, we could not find a prior study on gut mycobiome in people with CF evaluating the effects of ETI, and further studies are needed elucidate this.

The *Staphylococcus aureus* infection module, benzoate and ethylbenzene degradation pathways were significantly enriched in CF children not treated with ETI when compared to controls. The other analyzed pathways were enriched in healthy controls (Figure 12). Despite considerable methodological heterogeneity across prior studies, CF gut dysbiosis was characterized by delayed microbial maturation, and alteration in microbial functionalities such as enrichment of mucin/glycan degradation, enhancement of xenobiotic and antibiotic-associated pathways, increased *Staphylococcus aureus* infection modules, alteration in glycosphingolipid metabolism, and attenuation of short-chain-fatty-acid pathways [9,14,25,41]. *Staphylococcus aureus* infection module (a known respiratory pathogen in the CF) was more abundant in CF children not treated with ETI than in the other two groups, which could suggest susceptibility to this infection in CF children who did not receive ETI [41]. Reasoner et al. noted a decrease in *Staphylococcus aureus* in abundance in the gut following ETI therapy [34]. Benzoate and ethylbenzene degradation (representing xenobiotic metabolism) were more enriched in CF children not treated with ETI, and this is possibly linked to enhanced gut microbial detoxification processes [27]. Similarly, Fouhy et al. noted the results showed that there were significantly increased abundances of pathways involved in xenobiotic metabolism in the CF gut microbiota compared to the controls [42].

Also, the protein processing in the endoplasmic reticulum and glycosphingolipid biosynthesis pathways were higher (even though the differences were not statistically significant) in CF children treated with ETI compared to children not treated with ETI. Similarly, children with CF treated with ETI occupied an intermediate status between the healthy control group and children with CF who did not receive ETI. This could indicate potential metabolic shifts due to treatment effects [27]. Similarly, Reasoner et al. noted changes in functional microbial pathways, such as reduced abundance of microbiome-encoded antibiotic resistance genes, microbial pathways for aerobic respiration, and microbial acid tolerance genes, indicating microbial adaptation [34]. Similarly to our observation, prior studies in other vertebrates have noted prominent shifts in functional metabolic pathways in the gut microbiome influenced by different diets and environments [53,54].

There are several limitations in our study. The small sample size limits the generalization of the results. Also, we used a cross-sectional methodology instead of a conventional longitudinal design (comparing gut microbiome pre-ETI and post-ETI and evaluating the microbial changes over time) reported by other investigators [10,31,34,44]. We did not evaluate the respiratory details (*Staphylococcus aureus* and *Pseudomonas aeruginosa* carrier statuses), quality of diet, and nutritional status, which definitely could influence the microbiome [55].

We excluded patients who had recently used systemic antimicrobials (other than azithromycin) in the past twelve weeks, and this strict exclusion criterion prevented us from using the longitudinal design, as children with CF often require systemic antibiotics. However, this strict exclusion criteria helped us to minimize many confounders for the evaluation of dysbiosis. We also did not perform contamination assessment and negative controls which might have influenced the validity of the studies.

Our study has other notable strengths. We included gut mycobiome and functional analysis of the microbiome in the CF population post-ETI, where the literature remains sparse. The current pervasive use of ETI would allow for much larger studies, and this study may serve as a road map, providing important preliminary data. Future studies are needed to confirm or refute our preliminary findings. While the present work focuses on community-level functions, future taxon-stratified comprehensive functional analyses may link pathways to the specific bacterial and fungal taxa that drive them. Similarly, future studies evaluating the inter-kingdom interactions between bacteria and fungi also need to be further elucidated.

## 4. Materials and Methods

### 4.1. Study Design

This single-center, prospective, observational study was performed at the UH Rainbow Babies and Children’s Hospital, Cleveland. Pediatric patients with CF between 2 and 17 years were recruited from the CF multidisciplinary clinic between November 2021–April 2024.

### 4.2. Inclusion Criteria

Patients with CF who received the ETI for at least 2 months were included in the ETI group, and CF patients who did to receive ETI were included in the CF control group. The initiation of ETI was decided by their primary CF pulmonologist of the patient and was not influenced by the research team and, hence, this was not a clinical trial. We also recruited healthy siblings of CF patients in the same age group. Demographic and clinical information were obtained from the medical records.

### 4.3. Exclusion Criteria

Patients who had recently used systemic antimicrobials (other than azithromycin) in the past twelve weeks were excluded [55]. Any underlying concomitant immunodeficiency disorders, malignancy, autoimmune conditions, gastrointestinal infections, and mucosal gastrointestinal conditions were also excluded. Patient on CF-related metabolic syndrome.

Patients on acid suppression medications and laxatives were not excluded, as these medications were commonly prescribed in the selected patient population. We did not calculate the sample size given the exploratory nature of this pilot study.

### 4.4. Sample Collection and Processing

The stool samples were collected by parents/legal guardians, who were provided with a stool collection kit (BD BBL™ CultureSwab™ EZ, Franklin Lakes, NJ, USA) during enrollment along with instructions to return. Specimens were collected and stored in a consistent way to minimize confounding effects. All collected stool samples were instantly placed in FastPrep^®^ tubes (MP Biomedicals™, Cat# 5076-200-34340, Solon, OH, USA) containing 500 μL of glass beads (Sigma-Aldrich G8772-100g, St. Louis, MO, USA) and 1 mL ASL ™ lysis buffer (Qiagen DNA Extraction Kit, Hilden, Germany). To minimize the batch effect, all samples were stored at −20 °C, then processed and analyzed concurrently for microbial composition. The methodology was further detailed in the Supplementary Materials and was previously reported by us in our prior studies [56,57].

### 4.5. Bioinformatics

Demultiplexed reads were processed in QIIME 2 (v2024.10.1) [58]. DADA2 (v2024.10.0) [59] denoised the data to amplicon sequence variants (ASVs), with quality filtering (trimming bases with Phred < Q35), dereplication, and chimera removal. Taxonomy was assigned against SILVA 138 SSU [60] using the QIIME 2 feature-classifier, and species-level labels were refined by querying ASV representative sequences with NCBI BLAST+ (v2.17.0) [61] against the NCBI 16S/nt reference database. An approximately maximum-likelihood phylogeny was inferred with FastTree 2 (v2.1.11) [62]. Before creating the phyloseq object [63] for the 16S data, the taxonomic table was evaluated. Taxa annotated as “unidentified” or lacking species-level labels were retained for all analyses but excluded only in the generation of taxonomy bar plots to improve visualization clarity. With regard to total read counts per sample, a cutoff of 500 read counts was utilized as the minimum needed read count. In the final results, we obtained 642 ASVs, and among these ASVs there are 624 ASVs in the bacterial kingdom. Rarefaction depth: We evaluated sequencing depth sufficiency and the effect of rarefaction thresholds using rarefaction curves (Supplementary Figure S1). The curves show early saturation across samples, consistent with the low microbial richness characteristic of pediatric populations and especially of children with CF, who are known to exhibit delayed microbiome maturation and reduced bacterial diversity. This biological context explains the lower ASV count observed in our dataset (642 total; 624 bacterial), despite appropriate quality filtering. A rarefaction depth of 500 reads was selected to maximize sample retention for diversity analyses. Applying higher thresholds resulted in substantial sample loss in this relatively small cohort, which would reduce statistical power for group comparisons. All differential abundance analyses were conducted using unrarefied counts with DESeq2, following current best practices, ensuring that rarefaction did not influence differential abundance results.

We used the nomenclature as suggested by the National Center for Biotechnology Information (NCBI) [64]. For mycobiome analysis, demultiplexed reads were similarly processed in QIIME 2 (v2024.10.1) [58]. DADA2 (v2024.10.0). [59] denoised the data to ASVs, with quality filtering (trimming bases with Phred < Q35), dereplication, and chimera removal. For mycobiome, analysis of barcode-sorted samples was performed in a custom pipeline based on UNITE v7.2 database, illustrated for taxonomic classification of ITS sequences.

### 4.6. Statistical Analysis

For the clinical data, statistical analysis was performed using R v4.4.1. Fisher’s exact and Mann–Whitney U tests for statistical analysis; results with *p*-values less than 0.05 were deemed significant. Alpha and beta diversities, and the relative abundance analysis differences, were compared between groups. Alpha diversity was analyzed using 3 indices: the Shannon index, the Simpson index, and the observed features. For evaluating the relationship between alpha diversity and duration of ETI, the flexible regression method (generalized additive model) was used.

For beta diversity, the association between community composition and outcomes was assessed using permutation-based PERMNOVA [65] implemented using the vegan (v2.7.1) [66] R package with the function adonis. During the process of beta diversity analysis, distance matrices were computed using Bray–Curtis [67], unweighted UniFrac [68], and weighted UniFrac [69] methods. The differential analysis was implemented in R using LEfSe [70] and DESeq2 [71]. Non-parametric Spearman correlation and Wilcoxon rank-sum test were used for association with continuous outcome and binary outcomes, respectively. Longitudinal analysis was performed using all pairwise multiple comparisons of mean ranks as implemented in the PMCMR plus R package (v1.2.0), employing the Kruskal–Wallis [72] test followed by Bonferroni–Dunn post hoc adjustment [73]. A *p*-value < 0.05 was considered statistically significant for all tests after correcting for multiple comparisons. Correction for multiple tests was performed using the Benjamini–Hochberg adjustment [74]. Box plots were utilized to show the interquartile range (IQR) as a measure of statistical dispersion and the difference between the groups. The lines extending parallel to the boxes (whiskers) indicated variability outside the upper and lower quartiles, and the central line inside the box represented the median value. Functional prediction analysis was conducted using PICRUSt2-based inference implemented through the ggpicrust2 (v2.5.2) R workflow. Functional abundances were predicted at multiple pathway levels and subsequently normalized before statistical testing. For datasets involving more than two groups, ggpicrust2 (2.5.2) automatically performs pairwise differential analysis using DESeq2-based generalized linear models, allowing direct comparison between Disease-T, Disease-NT, and HC groups. This approach provides both effect sizes and adjusted *p*-values for each pairwise contrast rather than a single overall test. Multiple-testing correction was applied using the Benjamini–Hochberg method to reduce false discovery rate (FDR).

## 5. Conclusions

We did not find significant differences in the demographics and clinical characteristics between the three groups. *Firmicutes* and *Proteobacteria* were the most abundant phyla and core members across all samples, regardless of disease status or treatment. Alpha diversity was not significantly different between the groups for both bacteriome and mycobiome. ETI treatment appeared to partially restore the alpha diversity, but the differences between groups were not statistically significant. *Ascomycota* and *Basidiomycota* were the most abundant and core members across all samples, regardless of disease status or treatment. Alpha diversity showed a negative trend with the duration of ETI therapy for both bacteriome and mycobiome. Confirmation of these results in larger trials will provide further evidence of the impact of ETI on the gut microbiome.

## Figures and Tables

**Table 1:** Demographics and clinical characteristics.

Variables	CF with ETI Therapy	CF Without ETI Therapy	Non-CF Siblings	*p* Value
Total number	12	11	7	
Age (mean ± S.D) in years	5.15 ±1.92	3.89 ± 1.58	5.37 ± 1.3	0.12 *
Sex				0.21
Male	7	8	2	
Female	5	3	5	
F508del homozygosity			NA	0.99
Yes	6	5		
No	6	6		
EPI			UK	0.47
Yes	12	10		
No	0	1		
ETI duration (mean ± S.D)	5.3 ± 2.6	NA	NA	NA
(in months)	(range 2–10)			
Enteral feeds			NA	0.37
Yes	5	2		
No	7	9		
Immobilized lipase cartridge use			NA	0.37
Yes	5	2		
No	7	9		
Proton pump inhibitors			NA	0.99
Yes	5	5		
No	7	6		
Azithromycin			NA	0.66
Yes	5	3		
No	7	8		
Meconium ileus			NA	0.21
Yes	0	2		
No	12	9		

(* *p* value between CF treated and not treated. CF—cystic fibrosis; EPI—exocrine pancreatic insufficiency; ETI—Elexacaftor–Tezacaftor–Ivacaftor; NA—not applicable; UK—unknown).

## Data Availability

The original contributions presented in this study are included in the article/Supplementary Materials. Further inquiries can be directed to the corresponding author.

## Supplementary Materials

The following supporting information can be downloaded at: https://www.mdpi.com/article/10.3390/ijms27020814/s1.
