# Supplementary material for: Comparison of Stool Microbiome in Children with Cystic Fibrosis Treated with and Without Elexacaftor–Tezacaftor–Ivacaftor—A Pilot Study
Source: Int J Mol Sci. 2026 Jan 14;27(2):814. doi: 10.3390/ijms27020814 (PMC12841031; doi:10.3390/ijms27020814)
Supplement: Supplementary file 1 [file ijms-27-00814-s001.zip › ijms-4026895-supplementary.pdf]

Methodology:

DNA Extraction: DNA was extracted using a QIA amp Fast DNA Stool Mini kit (Qiagen GmpH, Germany) per the manufacturer's instructions. Stool swabs were transferred to tubes with one ml of InhibitEX lysis buffer. Swabs were incubated for sixty minutes at 75°C and shaken using FastPrep 96 two times for 300 seconds each at 1800 RPM. Equal amounts of 100% ethanol and lysate were mixed in a collecting tube and passed through HiBind DNA Mini Columns (Omega Bio-tek, USA) with the collected DNA pellet eluted using 50 ul molecular grade water. The quality of the isolated genomic DNA was confirmed by gel electrophoresis and quantitated with the Qubit 2.0 instrument applying the Qubit dsDNA HS Assay (Life Technologies, USA). DNA samples were stored at -20C.

PCR Amplification: Amplification of microbial 16S and 5.8S rRNA genes were performed using 16S-515 (5′-GGA CTA CCA GGG TAT CTA ATC CTG-3′) and 16S-804 (5′-(TCC TAC GGG AGG CAG CAGT-3′), ITS1 (5′-(TCC GTA GGT GAA CCT GCG G-3′), and ITS4 (5′-TCC TCC GCT TAT TGA TAT GC-3′) primers, respectively The PCR mixture was comprised of Q5 High-Fidelity Master Mix (New England Bioinformatics) at a 1x concentration, along with a double volume of molecular grade water and 0.05 ul/mM each primer. Template DNA (100 ng) was added to each 50 ul reaction. Thermo-cycling conditions consisted of an initial denaturation step (3 minutes at 98ºC), followed by 30 cycles of denaturation (10 seconds at 98ºC), annealing (10 seconds at 55ºC for the 16S primers), extension (10 seconds at 72ºC), and a final extension step of 3 minutes at 72ºC. Ten µl of each PCR product were separated using gel electrophoresis on 1.5% agarose gel (containing 7 µg/ml ethidium bromide).

Library Preparation and Sequencing: The amplicon library was cleaned and barcoded, followed by emulsion PCR using Ion Torrent S5 Prime workflow according to the manufacturer's instructions (ThermoFisher Scientific, Waltham, MA, USA). Equal volumes of bacterial 16S rRNA amplicons were pooled, cleaned with AMPure XP beads (Beckman Coulter, CA, USA) to remove unused primers, and then exposed to end repair enzyme for 20 minutes at room temperature. After an additional AMPure clean-up, ligation was performed at 25ºC for 30 minutes using Ion Torrent P1 and a unique barcoded 'A' adaptor per pooled sample after AMPure removal of residual adaptors. All separate barcoded samples were then pooled in equal amounts (10 µl) and sizes selected for the anticipated 16S using Pippin Prep (Sage Bioscience, Beverly, MA, USA). The library was amplified for seven cycles and quantitated on StepOne qPCR instrument ahead of proper dilution to 300 pM going into IonSphere templating reaction on the Ion Chef. Library sequencing was completed on an Ion Torrent S5 sequencer (ThermoFisher Scientific, Waltham, MA, USA).

Rarefaction Depth and Sensitivity Analyses:

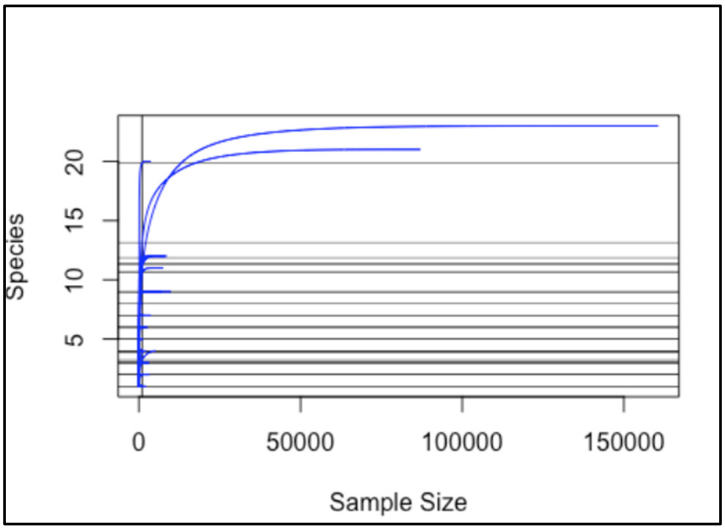

Supplemental Figure S1: Rarefaction curve for bacterial analysis

A rarefaction depth of 500 reads was selected to maximize sample retention, as applying a threshold of 5,000 reads removed 21 samples with insufficient sequencing depth. We also checked the distribution of per-sample sequencing depth distribution (**Supplemental Table S1**). Given the small cohort size (n = 30), retaining the samples was essential to preserve statistical power. We evaluated sequencing depth sufficiency and the effect of rarefaction thresholds using rarefaction curves (**Supplementary Figure S1**). The curves show early saturation across samples, consistent with the low microbial richness characteristic of pediatric populations and specifically in children with cystic fibrosis (CF), who are known to exhibit delayed microbiome maturation and reduced bacterial diversity. This biological context explained the lower amplicon sequence variants (ASV) count observed in our dataset (642 total; 624 bacterial), despite appropriate quality filtering.
A rarefaction depth of 500 reads was selected to maximize sample retention for diversity analyses. Applying higher thresholds resulted in substantial sample loss in this relatively small cohort, which would reduce statistical power for group comparisons. All differential abundance analyses were conducted using unrarefied counts with DESeq2, following current best practices, ensuring that rarefaction did not influence differential abundance results.

Statistic	Value
25th percentile (Q1)	2,093
Median	4,996
75th percentile (Q3)	11,135
IQR	9,043

Supplemental Table S1. Sequencing distribution for Bacteria

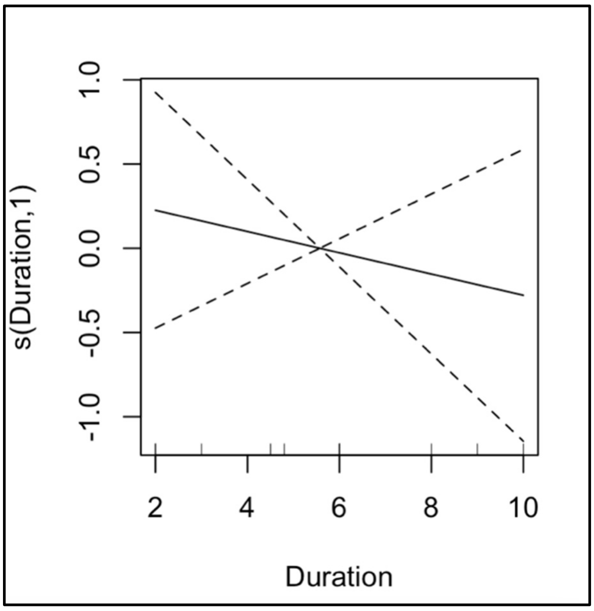

Supplemental Figure S2: Adjusted generalized additive model (GAM) for bacterial diversity

Abbreviations:

1. **Disease-NT** – Children with cystic fibrosis (CF) not treated with Elexacaftor-Tezacaftor-Ivacaftor (red color)
2. **Disease-T** – Children with CF treated with Elexacaftor-Tezacaftor-Ivacaftor (green color)
3. **HC** – Healthy sibling controls (blue color)

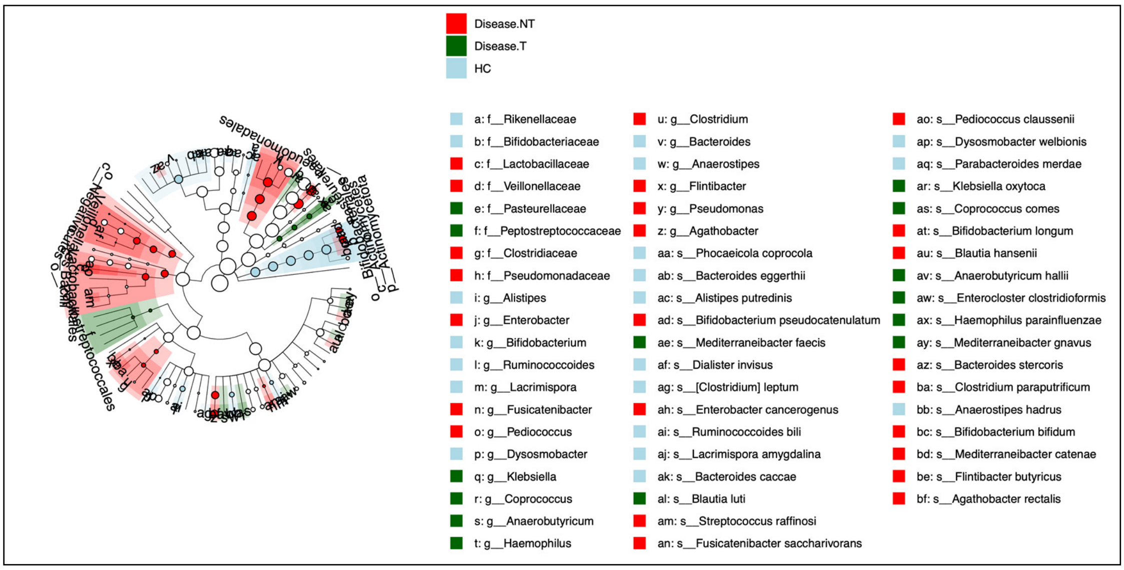

Supplemental Figure S3 – Cladogram of all-level significance using DESeq2 for bacteria

The following heatmap demonstrated the log10-transformed abundance of selected genera identified as significant through the LEfSe analysis (**Supplemental Figure S4**).

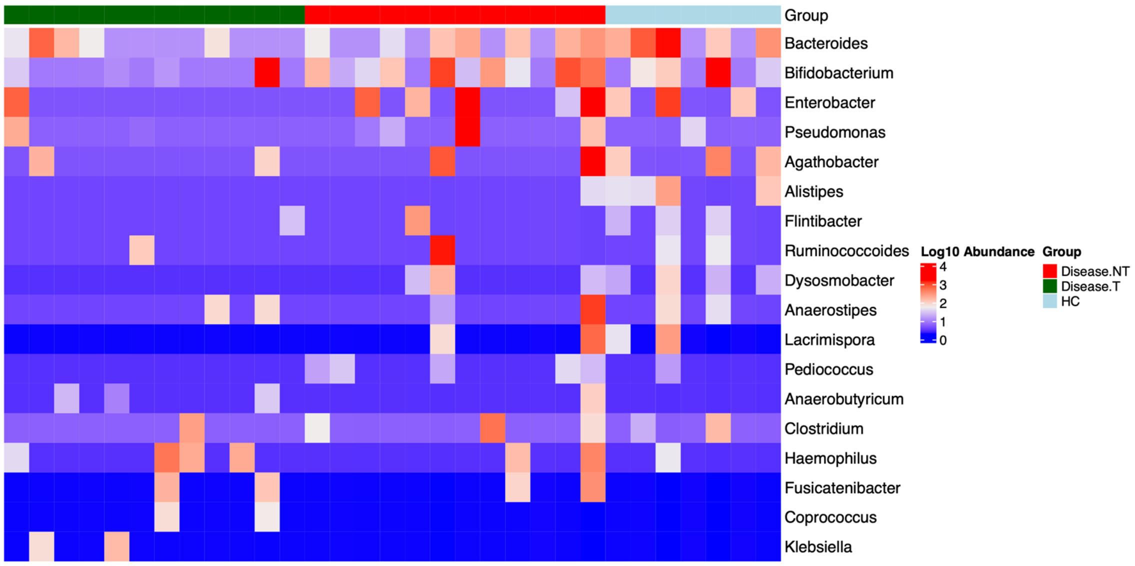

Supplemental Figure S4 - Genus level - DESeq2 (heat map) for bacteria

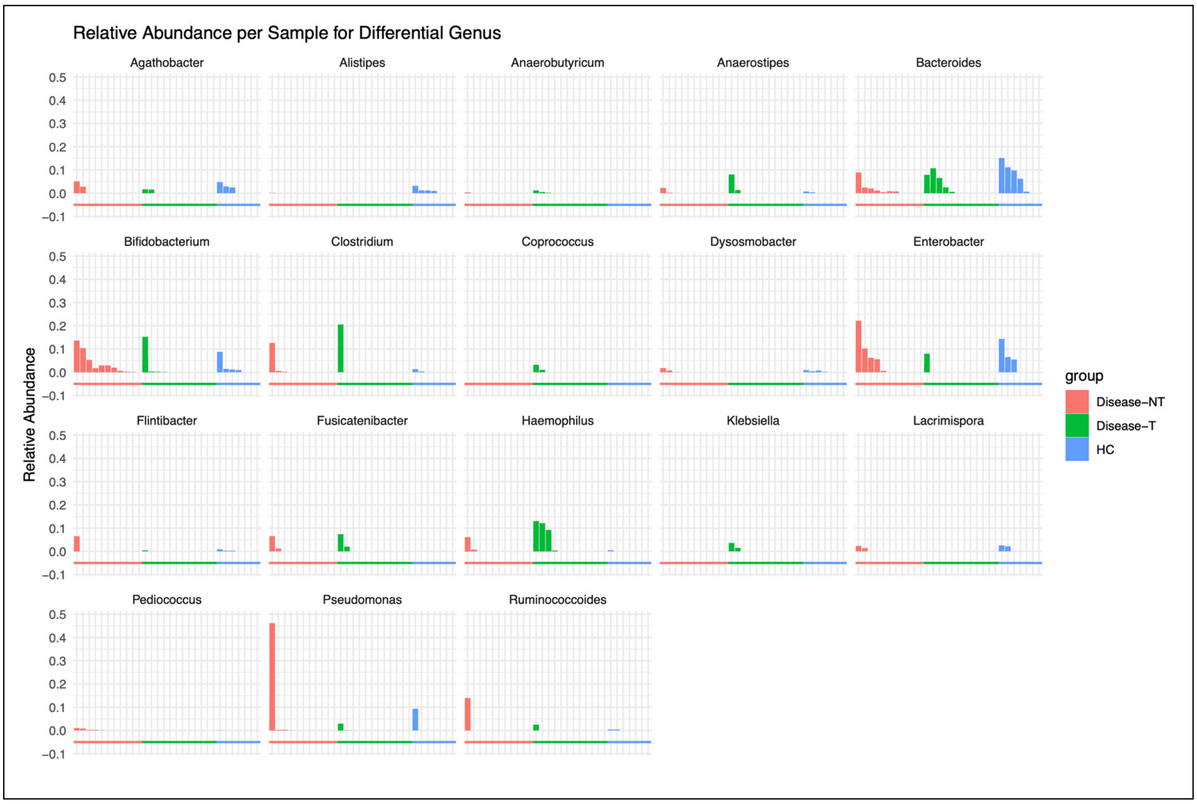

Supplemental Figure S5 - Genus – DESeq2 (bar plot) for bacteria

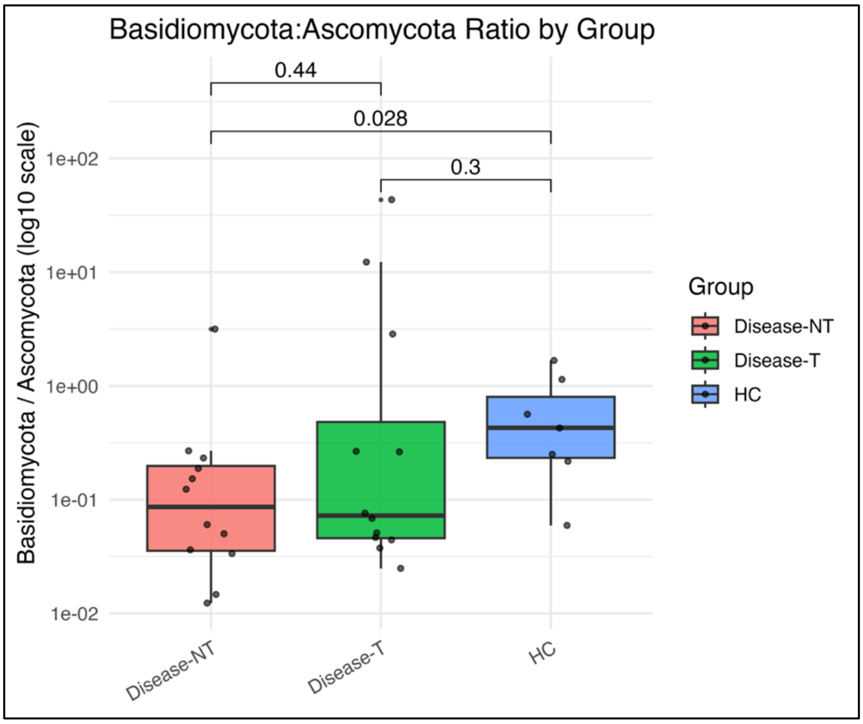

Supplemental Figure S6 - Basidiomycota/Ascomycota ratio in all three groups with p value

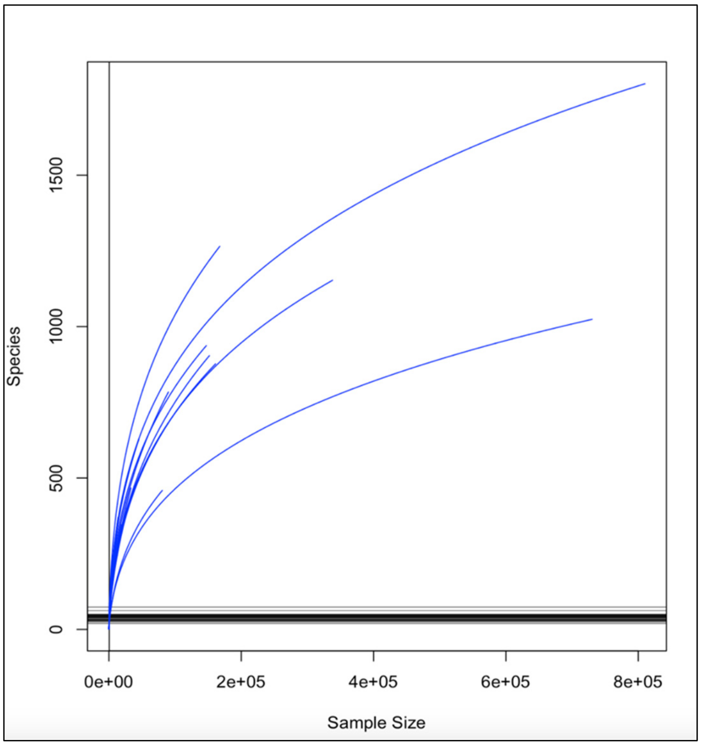

Supplemental Figure S7- Rarefaction curve for fungi

Statistic	Value
25th percentile (Q1)	1258

Median	13811
75th percentile (Q3)	85282
IQR	84024

Supplemental Table S2. Sequencing distribution for fungi

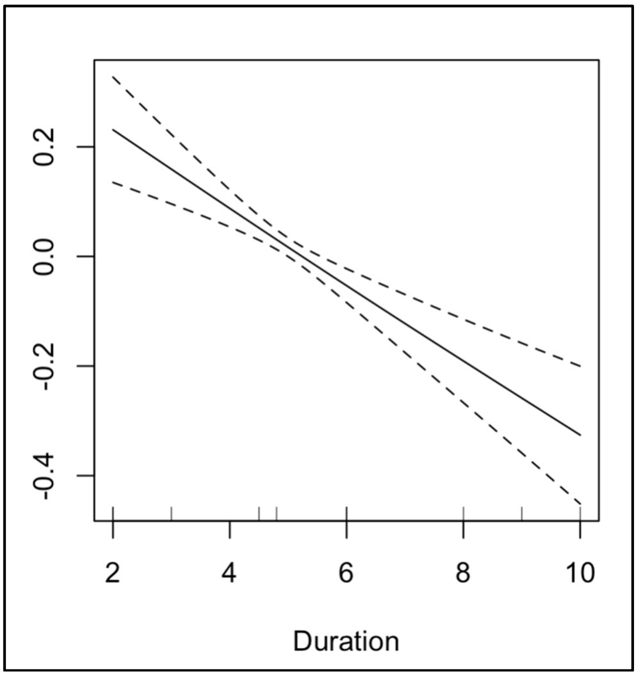

Supplemental Figure S8: Adjusted GAM model with duration of therapy (in months) for fungi

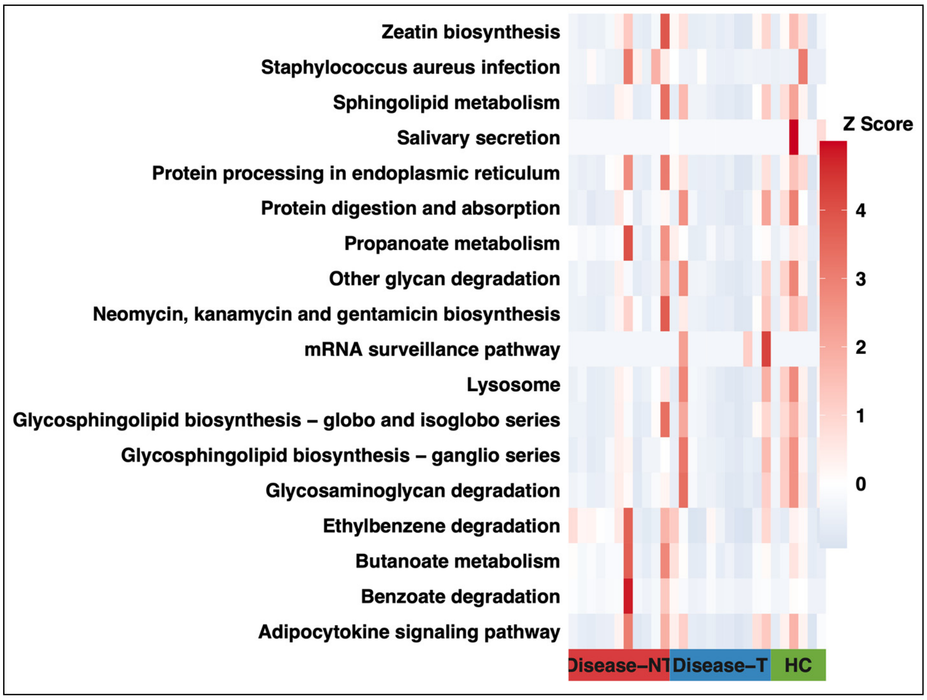

Supplemental Figure S9: Functional differences between the three groups (noted in z scores)

Comparison. Between Disease-NT and HC				
**Feature**	**Method**	**p_values**	**Pathway_name**	**Increased in**
ko04141	limma voom	0.029122077	Protein processing in endoplasmic reticulum	HC
ko00604	limma voom	0.000116538	Glycosphingolipid biosynthesis - ganglio series	HC
ko04974	limma voom	0.043400837	Protein digestion and absorption	HC
ko00531	limma voom	0.024113951	Glycosaminoglycan degradation	HC
ko00362	limma voom	0.010301501	Benzoate degradation	Disease-NT
ko00603	limma voom	0.022497815	Glycosphingolipid biosynthesis - globo and isoglobo series	HC
ko03015	limma voom	0.04935435	mRNA surveillance pathway	HC
ko00642	limma voom	0.046453685	Ethylbenzene degradation	Disease-NT
ko05150	limma voom	0.009176419	Staphylococcus aureus infection	Disease-NT

ko00511	limma voom	0.009318089	Other glycan degradation	HC
Comparison. Between Disease-T and HC				
ko04141	limma voom	0.103279927	Protein processing in endoplasmic reticulum	HC
ko00604	limma voom	0.081172414	Glycosphingolipid biosynthesis - ganglio series	HC
ko04974	limma voom	0.45506547	Protein digestion and absorption	HC
ko00531	limma voom	0.045473736	Glycosaminoglycan degradation	HC
ko00362	limma voom	0.203693818	Benzoate degradation	Disease-T
ko00603	limma voom	0.270293157	Glycosphingolipid biosynthesis - globo and isoglobo series	HC
ko03015	limma voom	0.147119728	mRNA surveillance pathway	Disease-T
ko00642	limma voom	0.086654368	Ethylbenzene degradation	Disease-T
ko05150	limma voom	0.235926573	Staphylococcus aureus infection	HC
ko00511	limma voom	0.199552727	Other glycan degradation	HC
Comparison. Between Disease-NT and Disease T				
ko04141	limma voom	0.709441595	Protein processing in endoplasmic reticulum	Disease-T
ko00604	limma voom	0.080496716	Glycosphingolipid biosynthesis - ganglio series	Disease-T
ko04974	limma voom	0.101504256	Protein digestion and absorption	Disease-T
ko00531	limma voom	0.408030346	Glycosaminoglycan degradation	Disease-T
ko00362	limma voom	0.024852941	Benzoate degradation	Disease-NT
ko00603	limma voom	0.233745278	Glycosphingolipid biosynthesis - globo and isoglobo series	Disease-T

ko03015	limma voom	**0.009353608**	mRNA surveillance pathway	Disease-T
ko00642	limma voom	**0.45956742**	Ethylbenzene degradation	Disease-NT
ko05150	limma voom	**0.131534567**	Staphylococcus aureus infection	Disease-NT
ko00511	limma voom	**0.235196266**	Other glycan degradation	Disease-T

Supplemental Table S3. Functional differences between the three groups
